# Therapeutic implication of HER2 in advanced biliary tract cancer

**DOI:** 10.18632/oncotarget.11157

**Published:** 2016-08-09

**Authors:** Ah-Rong Nam, Ji-Won Kim, Yongjun Cha, Hyerim Ha, Ji Eun Park, Ju-Hee Bang, Mei Hua Jin, Kyung-Hun Lee, Tae-Yong Kim, Sae-Won Han, Seock-Ah Im, Tae-You Kim, Do-Youn Oh, Yung-Jue Bang

**Affiliations:** ^1^ Cancer Research Institute, Seoul National University College of Medicine, Seoul, Korea; ^2^ Department of Internal Medicine, Seoul National University Bundang Hospital, Seongnam, Korea; ^3^ Department of Internal Medicine, Seoul National University Hospital, Seoul, Korea

**Keywords:** HER2, biliary tract cancer, gallbladder cancer, trastuzumab, targeted therapy

## Abstract

Currently, there is no validated therapeutic target for biliary tract cancer (BTC). This study aimed to investigate the pre-clinical and clinical implication of HER2 as a therapeutic target in BTC. We established two novel HER2-amplified BTC cell lines, SNU-2670 and SNU-2773, from gallbladder cancer patients. SNU-2670 and SNU-2773 cells were sensitive to trastuzumab, dacomitinib, and afatinib compared with nine HER2-negative BTC cell lines. Dacomitinib and afatinib led to G1 cell cycle arrest in SNU-2773 cells and apoptosis in SNU-2670 cells. Furthermore, dacomitinib, afatinib, and trastuzumab showed synergistic cytotoxicity when combined with some cytotoxic drugs including gemcitabine, cisplatin, paclitaxel, and 5-fluorouracil. In a SNU-2670 mouse xenograft model, trastuzumab demonstrated a good anti-tumor effect as a monotherapy and in combination with gemcitabine increasing apoptosis. In our clinical data, 13.0% of patients with advanced BTC were defined as HER2-positive. Of these, three patients completed HER2-targeted chemotherapy. Two of them demonstrated a partial response, and the other one showed stable disease for 18 weeks. In summary, these pre-clinical and clinical data suggest that HER2 could be a therapeutic target, and that a HER2-targeting strategy should be developed further in patients with HER2-positive advanced BTC.

## INTRODUCTION

In recent years, development of targeted agents significantly improved the prognosis of advanced cancer patients. However, there is no validated target for biliary tract cancer (BTC) as yet, and prognosis of advanced BTC patients is still dismal [1, 2]. Therefore, there is still huge unmet need for the development of novel therapeutic targets in advanced BTC.

HER2 overexpression or amplification is observed in approximately 10–16% of gallbladder cancer and 5–11% of extrahepatic bile duct cancer [3–5], although its biologic importance or clinical implication are not as well understood as in breast cancer and gastric cancer [6–8]. A previous study showed that constitutive expression of HER2 in gallbladder epithelium lead to the development of adenocarcinoma [9]. A cholangiocarcinoma cell line with high HER2 expression demonstrated enhanced invasiveness, motility, and proliferation compared with the other cell lines without HER2 overexpression via AKT/p70S6K pathway activation [10]. Therefore, it might be speculated that HER2 overexpression is important in the pathogenesis and progression of a certain subtype of BTC, leading to a dismal prognosis in this subset of patients as also observed in those patients with breast cancer with HER2 overexpression [11]. In addition, in gallbladder cancer, genes in the ErbB signaling pathways were more frequently mutated compared with other BTCs [12]. Therefore, it is suggested that overexpression or amplification of HER2 could be a promising therapeutic target in patients with BTC. HER2-targeted strategy has dramatically improved the clinical outcome of patients with breast and gastric cancer, and has already been incorporated into the standard clinical practice in these patients [6–8]. However, since not all genetic alterations in tumor tissues could be therapeutic targets, it is necessary to investigate the pre-clinical and clinical implication of HER2 overexpression or amplification as a therapeutic target in BTC.

On the basis of this background, we conducted pre-clinical and clinical studies to identify clinical implication of HER2 positivity and to provide evidence for HER2-targeted therapy in patients with advanced BTC.

## RESULTS

### Expression levels of HER family proteins in BTC cell lines

Two novel *HER2*-amplified human BTC cell lines, SNU-2670 and SNU-2773, were established from tumor tissues from BTC patients. SNU-2670 cell line was established from the metastatic liver mass of a 51-year-old female Asian patient (patient A; Table 1) with gallbladder adenocarcinoma harboring a *HER2* gene amplification with a HER2/CEP17 ratio of 5.76 by FISH and protein overexpression of 2+ by IHC. SNU-2773 cell line was derived from a metastatic neck lymph node of a 50-year-old male Asian patient (patient B; Table 1) with gallbladder adenocarcinoma harboring a *HER2* gene amplification with a HER2/CEP17 ratio of 2.67 by FISH and protein overexpression of 3+ by IHC.

**Table 1:** Characteristics of patients with HER2-positive BTC who completed trastuzumab-based chemotherapy

Patients	Sex/Age	Primary site	Metastasis	HER2 IHC	HER2 FISH	HER2-directed therapy	Best response	Duration of response	OS
A	F/51	Gallbladder	Liver and lymph nodes	2+	5.76	First-line: Trastuzumab + GP	SD	18 weeks	27 weeks
B	M/50	Gallbladder	Lung and lymph nodes	3+	2.67	Third-line: Trastuzumab + Paclitaxel Fourth-line: Novel Anti-HER2 Antibody	SD PR	25 weeks 34 weeks	Alive at 89 weeks
C	F/63	Gallbladder	Lymph nodes	3+	9.53	Third-line: Trastuzumab + XP	PR	12 weeks	79 weeks

Abbreviations: IHC, immunohistochemistry; FISH, fluorescent *in situ* hybridization; OS, overall survival; F, female; M, male; GP, gemcitabine/cisplatin; XP, capecitabine/cisplatin; SD, stable disease; PR, partial response.

**Figure 1:**
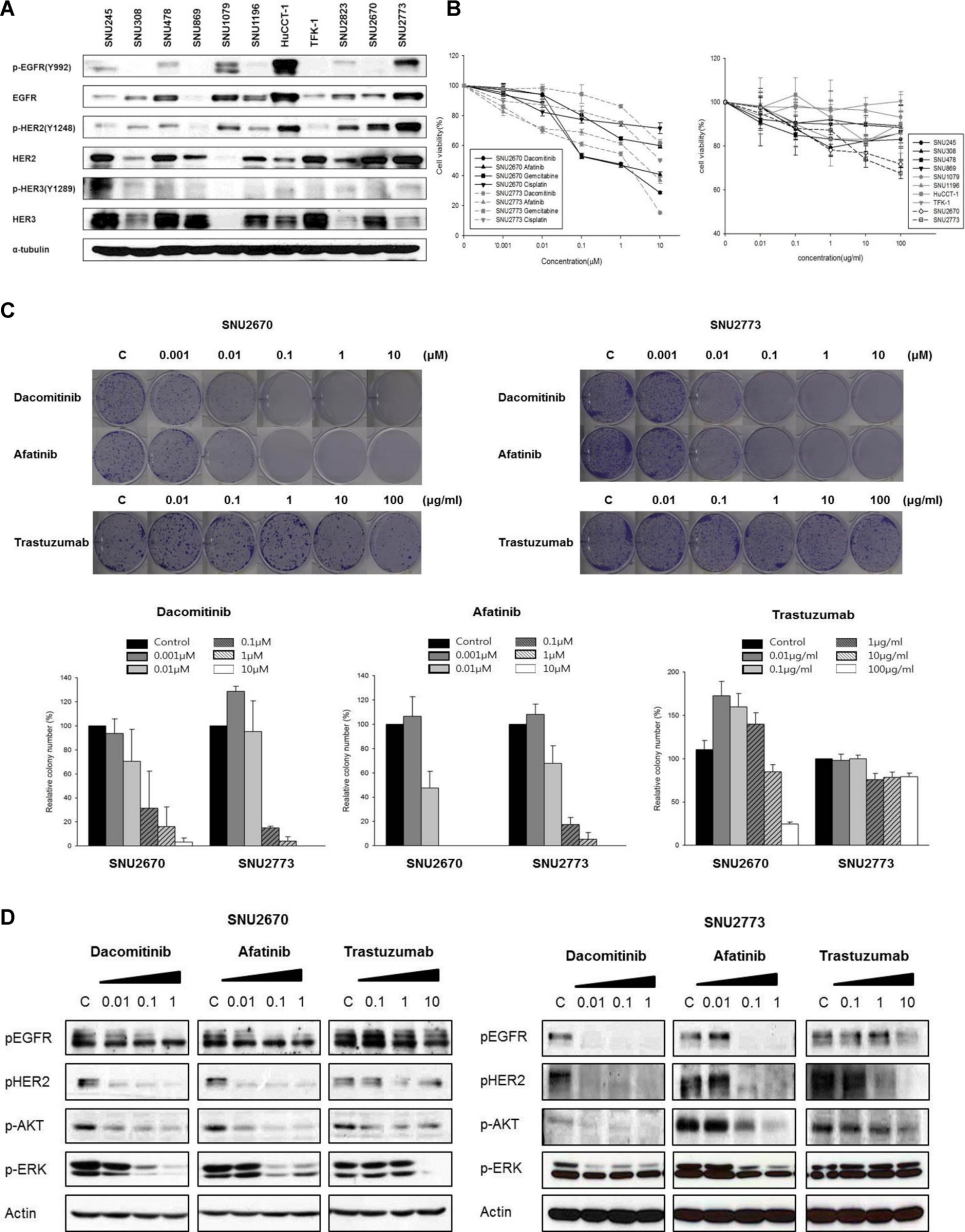
HER2-targeted treatment in *HER2*-amplified BTC cells (**A**) Both SNU-2670 and SNU-2773 cell lines showed strong HER2 expression compared with the other cell lines. (**B**) Using MTT assays, anti-proliferative effects of dacomitinib, afatinib, gemcitabine, and cisplatin were evaluated in SNU-2670 and SNU-2773 cells. Anti-proliferative effects of trastuzumab were evaluated in 10 human BTC cell lines: SNU-245, SNU-308, SNU-478, SNU-869, SNU-1079, SNU-1196, SNU-2670, SNU-2773, HuCCT1, and TFK-1. (**C**) Colony formation assays demonstrated the cytotoxic effect of dacomitinib, afatinib, and trastuzumab. (**D**) HER2-targeted agents abrogated downstream signaling pathways of HER2. The cells were treated with dacomitinib (0.01, 0.1, and 1 μM), afatinib (0.01, 0.1, and 1 μM), and trastuzumab (0.1, 1, and 10 μg/mL) for 48 h, and then immunoblotted with the indicated antibodies.

Among all 11 BTC cell lines, SNU-2670 and SNU-2773 expressed higher levels of total HER2 and phosphorylated HER2 compared with the other cell lines (Figure 1A). SNU-2670 and SNU-2773 cells harbored *HER2* gene amplification as measured by FISH, while SNU-245, SNU-308, SNU-478, SNU-869, SNU-1179, and SNU-1196 cells did not (Supplementary Figure S1). SNU2670 cells expressed high levels of HER3 and SNU2773 cells expressed high levels of EGFR compared to the other cell lines.

### Cytotoxic effects of various agents in HER2-amplified BTC cells

SNU-2670 cells were treated with various concentrations of targeted agents including trastuzumab, dacomitinib, and afatinib, and cytotoxic agents including gemcitabine, cisplatin, and 5-FU (Supplementary Table S1). Among targeted agents, dacomitinib and afatinib were the most sensitive drugs in SNU-2670 cells, with the half maximal inhibitory concentration (IC_50_) values of 0.10 μM and 0.13 μM, respectively. Both dacomitinib and afatinib also showed significant anti-proliferative effects in SNU-2773 cells with IC_50_ values of 1.5 μM and 1.6 μM, respectively (Supplementary Table S2). SNU-2670 and SNU-2773 cell lines were more sensitive to trastuzumab compared with other BTC cell lines (Figure 1B). The anti-proliferative effects of trastuzumab in these cells were comparable to those observed in HER2-positive gastric cancer cell lines, NCI-N87 and SNU-216, and a HER2-positive breast cancer cell line, SK-BR-3 [13]. Colony formation assays also showed significant inhibition of proliferation of HER2-amplified BTC cells (Figure 1C). In colony formation assays, the IC_50_ values of dacomitinib and afatinib were 0.045 μM and 0.010 μM in SNU-2670 cells and 0.042 μM and 0.019 μM in SNU-2773 cells, respectively.

### Downstream signaling pathways of HER2 after HER2-targeted therapy

Dacomitinib and afatinib decreased phosphorylation of EGFR and HER2 in SNU-2670 and SNU-2773 cells (Figure 1D). In addition, these targeted agents decreased phosphorylation of AKT, and ERK, two downstream molecules of EGFR and HER2. Trastuzumab abrogated phosphorylation of HER2 in SNU-2670 and SNU-2773 cells. In SNU-2670 cells, AKT and ERK phosphorylation was decreased by trastuzumab. In SNU-2773 cells, AKT phosphorylation was decreased by trastuzumab.

### Cell cycle arrest and apoptosis after HER2-targeted therapy

**Figure 2:**
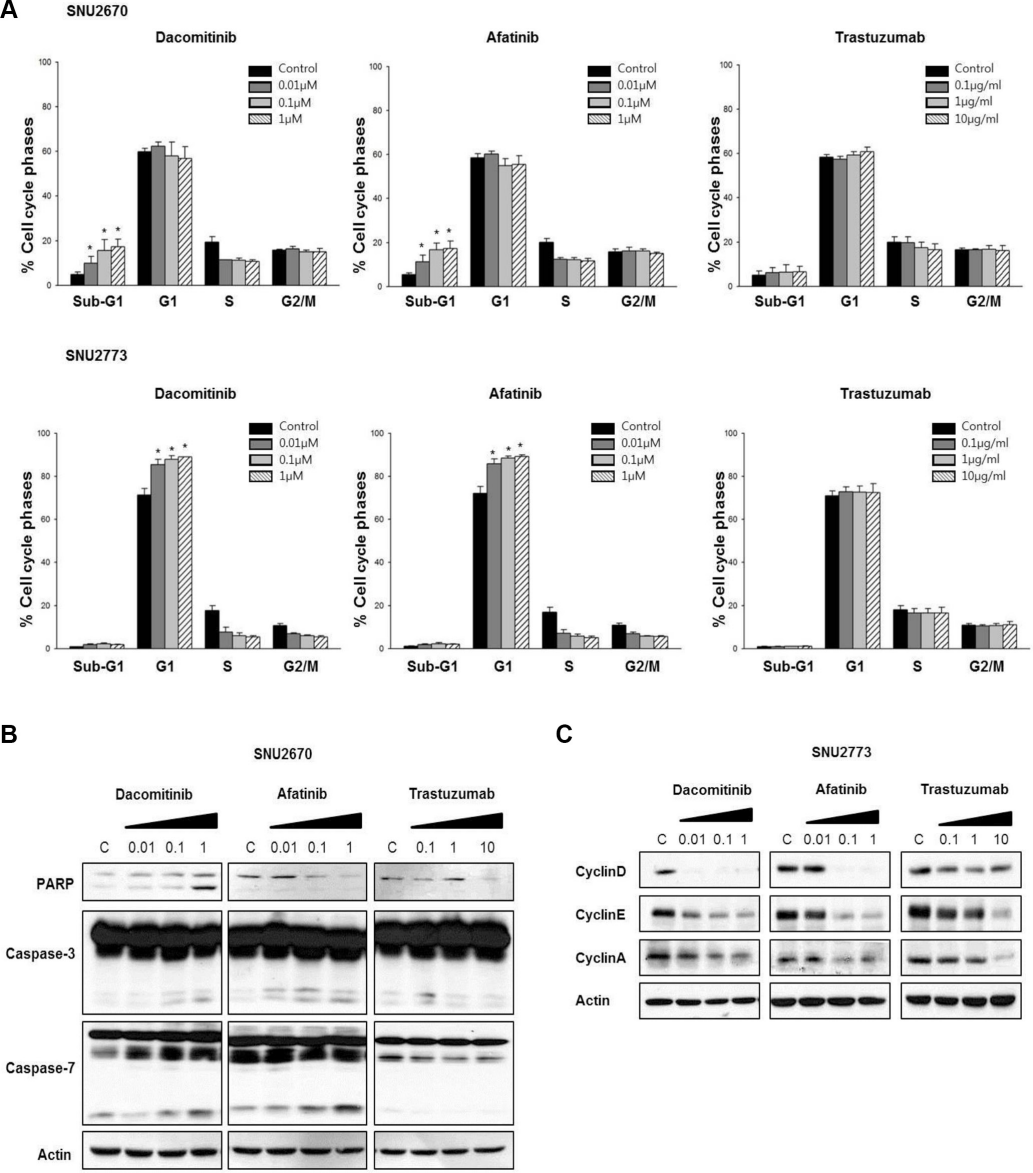
Cell cycle arrest and apoptosis after HER2-targeted therapy (**A**) Cell cycle analysis was performed in SNU-2670 and SNU-2773 cells after dacomitinib, afatinib, and trastuzumab treatment. *P < 0.05. (**B**) Effects of dacomitinib (0, 0.01, 0.1, and 1 μM), afatinib (0, 0.01, 0.1, and 1 μM), and trastuzumab (0, 0.1, 1, and 10 μg/mL) on apoptosis pathways were evaluated in SNU-2670 cells. (**C**) Effects of dacomitinib (0, 0.01, 0.1, and 1 μM), afatinib (0, 0.01, 0.1, and 1 μM), and trastuzumab (0, 0.1, 1, and 10 μg/mL) on G1 cell cycle arrest were evaluated in SNU-2773 cells.

Cell cycle analysis indicated that, in SNU-2670 cells, dacomitinib and afatinib increased sub-G1 population, while G1 arrest was more predominant in SNU-2773 cells. Trastuzumab tended to increase G1 arrest (Figure 2A). Dacomitinib increased cleavage of PARP, caspase-3, and caspase-7, and afatinib increased cleavage of caspase-3 and caspase-7 in SNU-2670 cells (Figure 2B). In SNU-2773 cells, dacomitinib and afatinib decreased cyclin D/E/A expression, and trastuzumab decreased cyclin E/A expression (Figure 2C).

### Synergistic effects of HER2-targeted agents and cytotoxic agents

**Figure 3:**
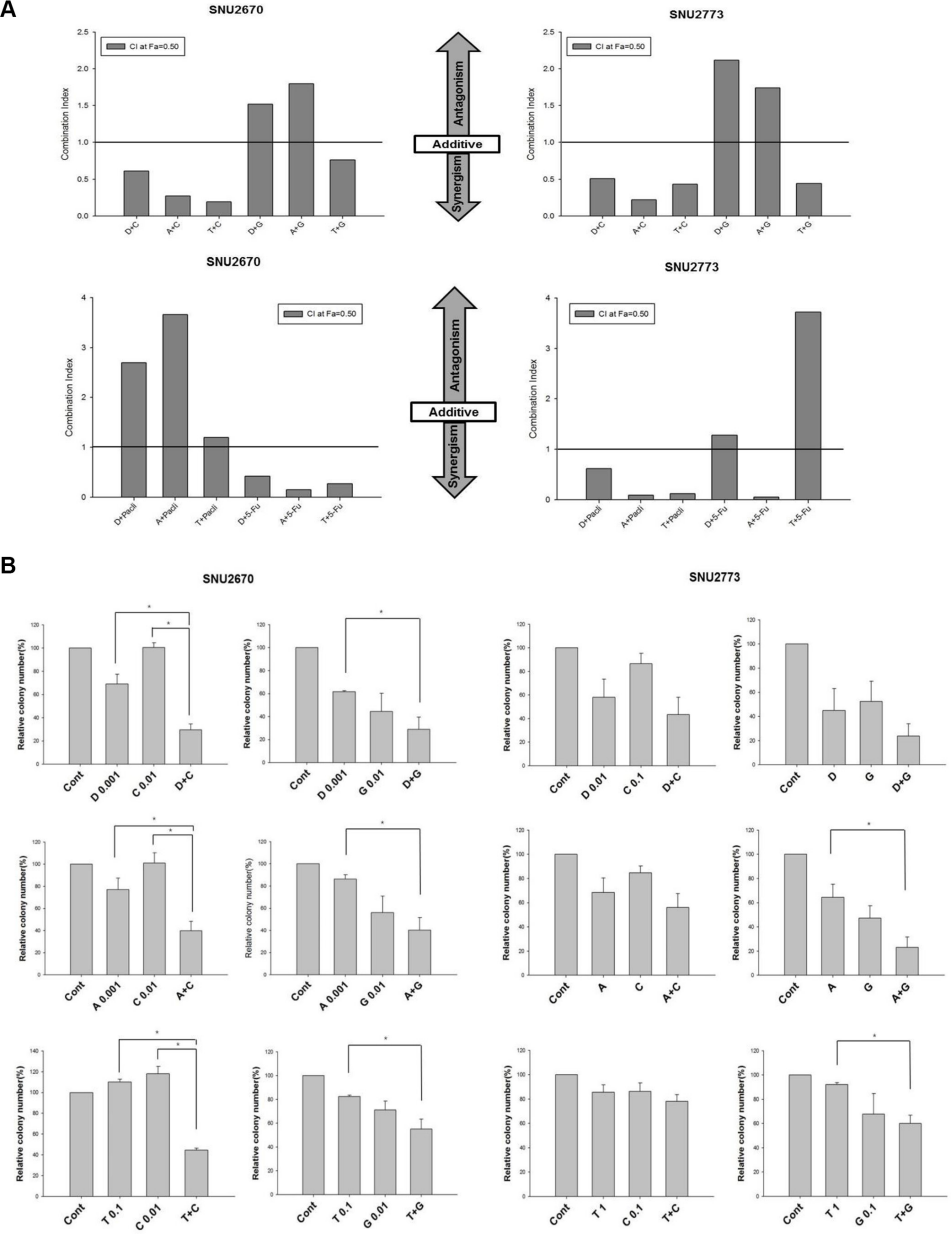

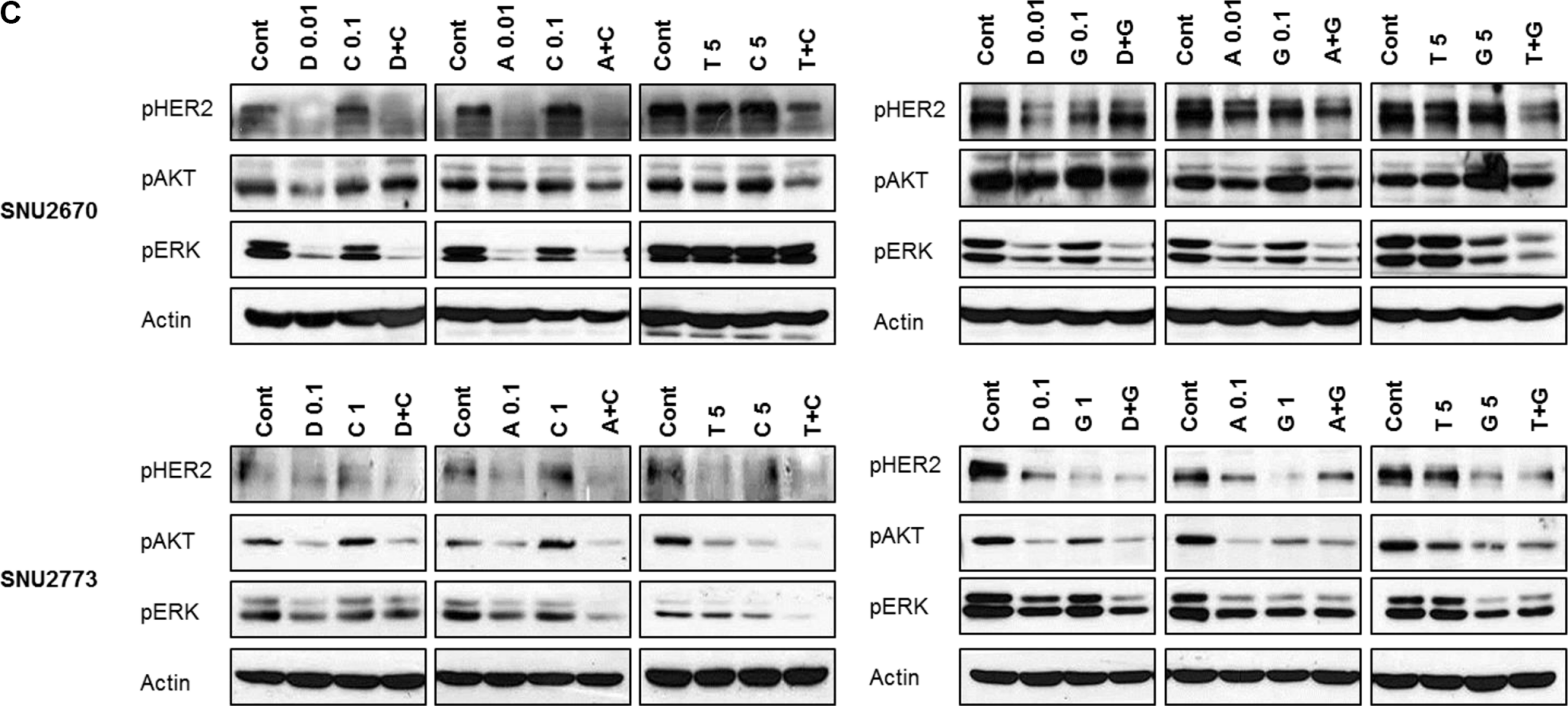
Synergistic cytotoxic effects of HER2-targeted agents and cytotoxic agents (**A**) Anti-proliferative effects of combination chemotherapy of HER2-targeted agents including dacomitinib (‘D’), afatinib (‘A’), or trastuzumab (‘T’) and a cytotoxic agent including cisplatin (‘C’), gemcitabine (‘G’), paclitaxel (‘P’), or 5-FU (‘F’) were evaluated in SNU-2670 and SNU-2773 cells using MTT assays. (**B**) Colony forming assays were performed to demonstrate the combined effect of a HER2-targeted agent and a cytotoxic agent. The concentration of each drug is indicated in the graph. The unit of concentration for each drug is μM for dacomitinib, afatinib, cisplatin, and gemcitabine and μg/mL for trastuzumab. The concentration of each agent in combination treatment was the same as that in single treatment. Anti-proliferative effects of combination chemotherapy were statistically compared with those of single agent chemotherapy to evaluate the efficacy of combination therapy. *P < 0.05. (**C**) Western blot analysis indicated that combination treatment more potently abrogated the downstream signaling pathways of HER2.

SNU-2670 and SNU-2773 cells were simultaneously treated with cytotoxic agents, targeted agents, and combinations of cytotoxic drugs and targeted drugs using MTT assays and colony formation assays. A HER2-targeted agent was administered in combination with a cytotoxic agent at a fixed ratio, which was decided on the basis of anti-proliferative effects of each drug (Figure 1B, Supplementary Tables S1 and S2). Dacomitinib or afatinib was combined with cisplatin, gemcitabine, paclitaxel, or 5-FU at a 1:10 ratio, and trastuzumab was combined with the cytotoxic agents at a 1 (μg/mL):1 (μM) ratio. In both SNU-2670 and SNU-2773 cells, dacomitinib and afatinib produced synergistic cytotoxicity in combination with cisplatin (combination index [CI] = 0.27 and 0.61 for SNU-2670 cells, and 0.51 and 0.22 for SNU-2773 cells, respectively; Figure 3A). In addition, in SNU-2670 cells, dacomitinib and afatinib showed synergistic anti-proliferative effects when combined with 5-FU (CI = 0.42 and 0.15, respectively). In SNU-2773 cells, dacomitinib synergized the anti-proliferative effects of paclitaxel (CI = 0.62), and afatinib synergized both paclitaxel and 5-FU (CI = 0.09 and 0.05, respectively). Trastuzumab also showed synergistic cytotoxic effects when combined with either gemcitabine or cisplatin in the two cell lines (CI = 0.76 and 0.19 for SNU-2670 cells, and 0.44 and 0.49 for SNU-2773 cells, respectively). In SNU-2670 cells, trastuzumab combined with 5-FU resulted in synergistic anti-proliferative effect (CI = 0.27). In SNU-2773 cells, trastuzumab plus paclitaxel demonstrated synergism (CI = 0.12). The same findings were reproduced in colony formation assays (Figure 3B and Supplementary Figure S2): combination of a targeted agent and a cytotoxic agent demonstrated significantly decreased cell proliferation than treatment with a targeted agent alone. In western blot analysis, dacomitinib or afatinib combined with cisplatin abrogated phosphorylation of HER2 and its downstream molecules, AKT and ERK (Figure 3C). In SNU-2670 cells, dacomitinib or afatinib combined with gemcitabine decreased ERK phosphorylation. In SNU-2773 cells, dacomitinib and gemcitabine abrogated phosphorylation of HER2, AKT, and ERK. Trastuzumab in combination with cisplatin or gemcitabine decreased HER2 phosphorylation. Downregulation of AKT and ERK was also observed in cells treated with trastuzumab plus cisplatin or gemcitabine.

### Anti-tumor effects of trastuzumab alone or in combination with gemcitabine in xenograft model

**Figure 4:**
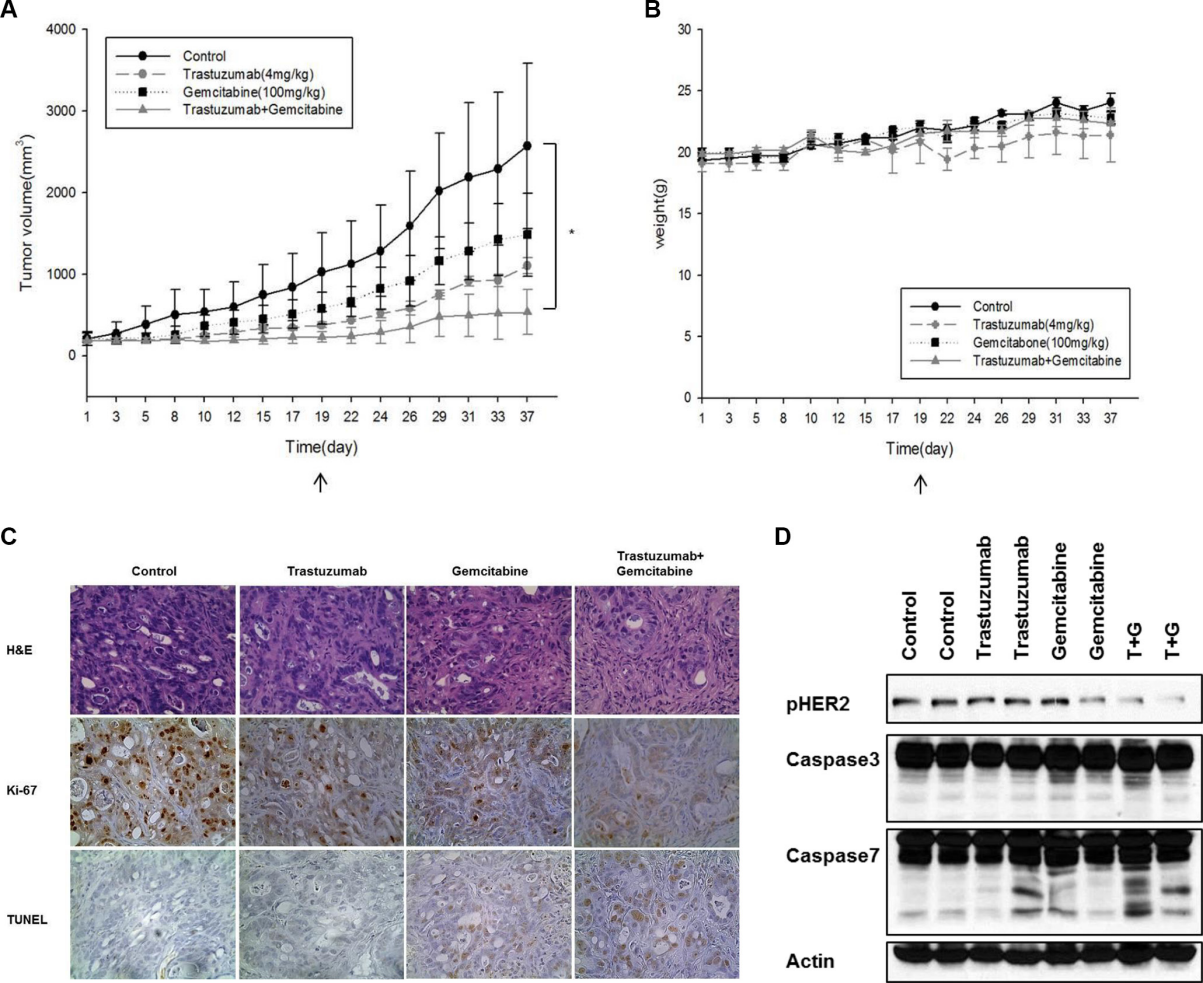
Synergistic anti-tumor effects of trastuzumab combined with gemcitabine in a mouse xenograft model (**A**) *In vivo* efficacy of trastuzumab alone, gemcitabine alone, or a combination of the two drugs was evaluated using a SNU-2670 xenograft model. *P < 0.05. (**B**) To evaluate the effect of chemotherapy on body weight, the body weight of mice was measured every other day. (**C**) At 37 days the tumors were harvested and analyzed using IHC. Ki-67 and TUNEL assays were performed to evaluate cell proliferation and apoptosis, respectively. (**D**) Western blot assays were performed using the excised tumors from the xenograft model to elucidate the effect of trastuzumab and/or gemcitabine on HER2 phosphorylation and apoptosis molecules.

We confirmed the *in vivo* efficacy of trastuzumab alone, gemcitabine alone, or a combination of the two drugs in a SNU-2670 xenograft model. Trastuzumab alone showed significant antitumor activity, and the combination of trastuzumab and gemcitabine significantly enhanced antitumor activity (*P* = 0.020) (Figure 4A). In addition, combination treatment did not influence the body weight of mice (Figure 4B). Tumors treated with trastuzumab alone or gemcitabine alone exhibited a significant decrease in cell proliferation by Ki-67 assays and a significant increase in apoptosis by terminal deoxynucleotidyl transferase–mediated dUTP nick end labeling (TUNEL) assays. When the tumors were treated with a combination of trastuzumab and gemcitabine, cell proliferation was further decreased and apoptosis was further increased (Figure 4C). Western blot analysis demonstrated that trastuzumab or gemcitabine alone increased cleavage of caspase-7 (Figure 4D). The combination of trastuzumab and gemcitabine markedly decreased HER2 phosphorylation and increased cleavage of caspase-7.

### Clinical implication of HER2-positivity in advanced BTC patients

Between July 2013 and July 2015, a total of 46 patients were enrolled. The baseline characteristics are listed in Table 2.

**Table 2:** Baseline characteristics of patients

Characteristics (*N* = 46)		Values
Age - years	Median (range)	59 (31–78)
Sex – No. (%)	Male	25 (54.3)
	Female	21 (45.7)
Primary site – No. (%)	Intrahepatic cholangiocarcinoma	11 (23.9)
	Extrahepatic cholangiocarcinoma	11 (23.9)
	Gallbladder cancer	21 (45.7)
	Ampulla of Vater cancer	3 (6.5)
Prior treatments before palliative first-line chemotherapy – No. (%)	Curative surgery	15 (32.6)
	Adjuvant chemoradiotherapy and/or chemotherapy	7 (15.2)
	Palliative surgery	13 (28.3)
	Palliative chemoradiotherapy and/or radiotherapy	7 (15.2)
No. of metastatic sites – No. (%)	1	17 (37.0)
	2	16 (34.8)
	3	10 (21.7)
	≥ 4	3 (6.5)
Sites of metastasis – No. (%)	Liver	30 (65.2)
	Lymph node	29 (63.0)
	Peritoneum	11 (23.9)
	Lung	10 (21.7)
	Bone	5 (10.9)
	Others*	3 (6.5)
HER2 status – No. (%)	IHC 3+	5 (10.9)
	IHC 2+ and FISH-positive	1 (2.2)
	IHC 2+ and FISH-negative	5 (10.9)
	IHC 2+ and FISH unknown	2 (4.3)
	IHC 1+	12 (26.1)
	IHC-negative	21 (45.7)
ECOG performance status – No. (%)	0 or 1	44 (95.7)
	2	1 (2.2)
	Unknown	1 (2.2)
First-line chemotherapy	Gemcitabine + Cisplatin	40 (87.0)
	Others†	6 (13.0)

* Others include adrenal gland (*N* = 2) and pleura (*N* = 1).

† Others include trastuzumab + gemcitabine + cisplatin (*N* = 2), gemcitabine + oxaliplatin (*N* = 1), gemcitabine + carboplatin (*N* = 1), TS-1 single (*N* = 1), and no treatment (*N* = 1).

Abbreviations: HER2, human epidermal growth factor receptor 2; IHC, immunohistochemistry; FISH, fluorescent *in situ* hybridization; ECOG, Eastern Cooperative Oncology Group.

Five of 46 patients (10.9%) were HER2 3+ by IHC: all had gallbladder cancer. Among eight patients with HER2 IHC 2+, HER2 FISH was performed in six patients and one (16.7%) was defined as FISH-positive: this patient also had gallbladder cancer. In summary, a total of six patients (13.0%) were defined as HER2-positive in our study cohort. In patients with gallbladder cancer (*N* = 21), the proportion of HER2-positive disease was 28.6%.

**Figure 5:**
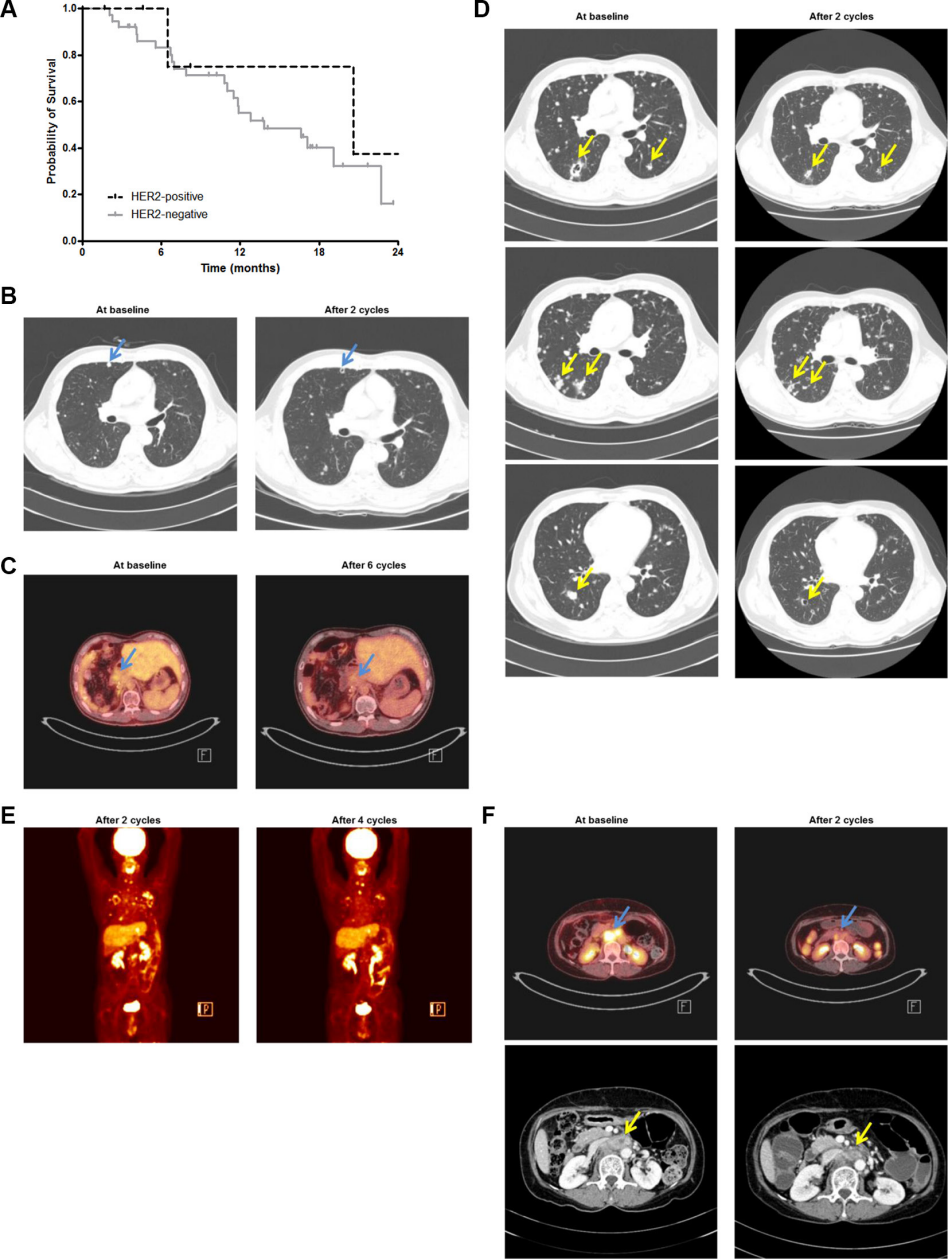
Clinical implications of HER2-targeted therapy in patients with advanced BTC (**A**) In our patient cohort, patients with HER2-positive disease tended to live longer than those with HER2-negative disease (median OS, 20.6 months *vs.* 13.8 months; *P* = 0.430). Two patients with HER2 IHC 2+ and FISH unknown were excluded from the survival analysis. (**B**) In Patient B, after two cycles of third-line trastuzumab/paclitaxel chemotherapy, CT scanning revealed a cystic change in the metastatic lung nodules (arrows). (**C**) After six cycles, ^18^F-fluorodeoxyglucose (^18^F-FDG) positron emission tomography scans revealed decreased maximal standardized uptake values from 3.4 to 2.2 (arrows). (**D**) After progression, the patient was enrolled in a phase I clinical trial of a novel anti-HER2-monoclonal antibody monotherapy as a fourth-line chemotherapy. After two cycles, CT scanning demonstrated a cystic change in the metastatic lung nodules (arrows). (**E**) After four cycles, ^18^F-FDG PET scanning revealed further decreased metabolism in metastatic tumors in the lungs, T1 spine, left adrenal gland, and lymph nodes. (**F**) In Patient C, after two cycles of third-line chemotherapy of trastuzumab combined with capecitabine and cisplatin, ^18^F-FDG PET scanning demonstrated markedly decreased metabolism in the retroperitoneal lymph node metastasis (arrows) with maximal SUV decrease from 13.98 to 7.3. In addition, CT scanning indicated that the size of the metastatic retroperitoneal lymph nodes (arrows) decreased from 36.2 mm × 21.7 mm to 29.1 mm × 13.9 mm.

During a median follow-up of 17.6 months (range, 1.7–33.0 months), 37 patients (80.4%) experienced disease progression and 25 patients (54.3%) died. In this cohort, 45 patients (97.8%) received palliative chemotherapy. Treatment response to first-line chemotherapy was evaluated in 42 patients (91.3%). The response rate (RR) was 8.7% (*N* = 4). The median progression-free survival (PFS) for first-line chemotherapy was 4.7 months (95% confidence interval, 2.2–7.2 months). The median overall survival (OS) was 16.6 months (95% confidence interval, 9.9–23.3 months). PFS were not significantly different according to HER2 status (*P* = 0.747). Patients with HER2-positive disease tended to have longer OS numerically compared with those with HER2-negative disease without statistical significance (median OS, 20.6 months *vs.* 13.8 months; *P* = 0.430; Figure 5A).

### Clinical efficacy of HER2-targeted therapy in HER2-positive BTC patients

In the aforementioned cohort (*N* = 46), a total of four patients (8.7%) received HER2-targeted chemotherapy. Among them, three patients completed the treatment at the time of analysis. The baseline characteristics and treatment details of the three patients are summarized in Table 1.

Patient A was a 51-year-old female Asian who was diagnosed with gallbladder cancer with hepatic and lymph node metastasis. She received palliative first-line chemotherapy with trastuzumab combined with gemcitabine and cisplatin. The best response was stable disease (SD). A total of five cycles of gemcitabine and cisplatin were administered, along with three cycles of trastuzumab from cycles 3 to 5. The duration of response was 18 weeks. The patient died 27 weeks after the start of first-line chemotherapy.

Patient B was a 50-year-old male Asian diagnosed with gallbladder cancer whose disease progressed to the lymph nodes and lung at 7 months after palliative resection and concurrent chemoradiation followed by chemotherapy with 5-FU and leucovorin. He had failed two lines of palliative chemotherapy including gemcitabine/cisplatin (PFS, 32 weeks) and 5-FU/leucovorin (PFS, 4 weeks). The patient was treated with third-line chemotherapy consisting of trastuzumab/paclitaxel. After two cycles of chemotherapy, CT scanning identified a cystic change in the metastatic lung nodules (Figure 5B). At baseline and after six cycles of chemotherapy,^18^F-fluorodeoxyglucose (^18^F-FDG) PET revealed that the maximal standardized uptake values (SUVs) in the retroperitoneal lymph nodes (Figure 5C) and left supraclavicular lymph nodes decreased from 3.40 to 2.20 and from 1.93 to 1.70, respectively. The best response was SD. The PFS for third-line chemotherapy was 25 weeks. After progression, the patient was enrolled in a phase I clinical trial of a novel anti-HER2-monoclonal antibody monotherapy. After two cycles, CT scanning identified a cystic change in the metastatic lung nodules (Figure 5D). After four cycles, ^18^F-FDG PET scanning revealed further decreased metabolism in metastatic tumors in the lungs, T1 spine, left adrenal gland, and lymph nodes (Figure 5E). The best response was partial response (PR). The PFS for fourth-line chemotherapy was 34 weeks. Nevertheless, he remained alive at 89 weeks after third-line chemotherapy.

Patient C was a 63-year-old female Asian diagnosed with gallbladder cancer with lymph node metastasis, which harbored *HER2* gene amplification with a HER2/CEP17 ratio of 9.53 by FISH (Supplementary Figure S3) and was HER2 3+ by IHC. After palliative resection, she had failed two lines of palliative chemotherapy. Then, she received palliative third-line chemotherapy of trastuzumab combined with capecitabine and cisplatin. After two cycles, ^18^F-FDG PET scanning demonstrated markedly decreased metabolism in the retroperitoneal lymph node metastasis with maximal SUV decrease from 13.98 to 7.3 (Figure 5F). In addition, CT scanning indicated that the size of the metastatic retroperitoneal lymph nodes decreased from 36.2 mm × 21.7 mm to 29.1 mm × 13.9 mm. The best response was PR. The PFS was 12 weeks. The patient died 79 weeks after the start of first-line chemotherapy.

## DISCUSSION

Available lines of evidence suggest that HER2 could be used for a novel therapeutic target in patients with BTC. However, preclinical evidence of HER2-directed therapy in *HER2*-amplified BTC cells has not yet been reported. In addition, only a few anecdotal cases have been published on the promising anti-tumor activity of trastuzumab-based combination chemotherapy in patients with HER2-positive BTC [14–16]. In this study, we successfully established two *HER2*-amplified BTC cell lines, SNU-2670 and SNU-2773, from patients with *HER2*-amplified BTC. In both *in vitro* and *in vivo* experiments using these cells, HER2-targeted agents such as dacomitinib, afatinib, and trastuzumab potently inhibited proliferation via abrogation of downstream signaling pathways of HER2, leading to G1 cell cycle arrest and apoptosis. In addition, we revealed that trastuzumab could be effectively combined with conventional cytotoxic agents including gemcitabine and cisplatin, which is currently the standard treatment option in patients with advanced BTC in the first-line setting [17]. In our patient cohort, three patients with HER2-positive BTC completed HER2-targeted chemotherapy, and clinical anti-tumor activity was observed in all of them: two patients demonstrated a partial response, and the other one showed stable disease for 18 weeks.

In our *in vitro* study, dacomitinib could nearly completely inhibit HER2 phosphorylation in both SNU-2670 and SNU-2773 cell lines. In addition, dacomitinib and afatinib could not potently inhibit EGFR phosphorylation in SNU-2670 cells compared with SNU-2773 cells. Nevertheless, SNU-2670 cells were more sensitive to dacomitinib and afatinib than SNU-2773 cells: the IC_50_ values of SNU-2773 cells for dacomitinib and afatinib were higher more than 10 times compared with SNU-2670 cells. Because dacomitinib inhibits EGFR and HER4 as well as HER2, and afatinib inhibits EGFR as well as HER2, it is very difficult to estimate the amount of anti-proliferative effects of targeted agents merely on the basis of inhibitory effects of a single target molecule. Moreover, anti-proliferative effects of targeted agents were influenced by many factors such as target expression levels, mutational status, or protein stability of target molecules or down-stream molecules. Our results suggest that, in SNU-2670 cells, HER2 might be more important than EGFR when dacomitinib induced anti-proliferative effects, while both HER2 and EGFR were thought to be important in SNU-2773 cells.

In our *in vitro* study data, the anti-proliferative effects of HER2-targeted agents in *HER2*-amplified BTC cells were comparable to those of previous studies using HER2-positive gastric or breast cancer cell lines. In both SNU-2670 and SNU-2773 cells, the IC_50_ values of dacomitinib and afatinib were similar to those of SNU-216 and NCI-N87 cells [18]. In addition, the anti-proliferative effects of trastuzumab in both SNU-2670 and SNU-2773 cells were comparable to those observed in HER2-positive gastric or breast cancer cells [13]. Therefore, it is suggested that the clinical efficacy of HER2-targeted therapy in patients with HER2-positive BTC could be substantial compared to those in patients with HER2-positive gastric or breast cancer.

In our data, dacomitinib and afatinib appear to be more potent in terms of anti-proliferative effects than trastuzumab *in vitro*. In addition, trastuzumab plus cisplatin appear to be more synergistic than trastuzumab plus gemcitabine in SNU-2670 cells. Nevertheless, we performed *in vivo* experiments using trastuzumab plus gemcitabine combination using the SNU-2670 xenograft model. In the history of clinical development of HER2-targeted agents, the starting point was trastuzumab, which was followed by other HER2-targeted agents such as lapatinib, trastuzumab emtansine (T-DM1), pertuzumab, *etc.* Although HER2-targeted small molecule inhibitors demonstrated excellent *in vitro* anti-proliferative activity [13, 19], these small molecule inhibitors could not demonstrated superiority over trastuzumab in terms of clinical efficacy so far [20, 21]. In addition, gemcitabine had long been used as a backbone to treat patients with advanced BTC, and recent studies showed that gemcitabine plus cisplatin is superior to gemcitabine alone [17, 22, 23]. Because cisplatin induces cumulative dose-dependent toxicities such as nephropathy and neuropathy in clinical practice, it cannot be administered for a long time compared with gemcitabine in spite of clinical benefits. Therefore, we believe that gemcitabine plus trastuzumab might be the combination which could be exposed to patients with long-term tolerability.

Interestingly, in our study as well as previously published reports [14–16], all the responders to trastuzumab-based chemotherapy were gallbladder cancer patients. In previous studies, HER2 positivity of BTC differs according to its primary site: among BTCs, the proportion of HER2 3+ by IHC was highest in gallbladder cancer, followed by extrahepatic cholangiocarcinoma and intrahepatic cholangiocarcinoma [4, 5]. In our study, all six patients with HER2-positive disease had gallbladder cancer. In addition, recent genomic studies identified that the mutational profiles of various BTCs were quite distinct according to their primary sites or causative etiologies [12, 24–26]. These results suggest that the pathogenesis of BTCs may be distinct according to these factors as well, and thus different treatment strategies may be applicable according to the clinical information and the results of genetic or molecular profiling. Therefore, to increase the efficacy of chemotherapy in BTC patients, further studies should focus on the identification of potential biomarkers for targeted therapy, to enable the selection and enrichment of patients who can benefit more from novel targeted agents.

The prognostic role of HER2 overexpression or amplification has not yet been thoroughly investigated in BTC. Some studies failed to demonstrate the prognostic impact of HER2 overexpression by IHC in patients with BTC, in part because of the relatively small sample size and heterogeneity of patient characteristics [27, 28], while the other reports showed that HER2 overexpression or amplification indicated worse prognosis of patients [29, 30]. The prognostic role of HER2 overexpression or amplification is still inconclusive in our study, possibly because of the effects of HER2-directed therapy in these patients.

In conclusion, our pre-clinical and clinical data suggest that HER2-directed therapy can be a promising treatment option in patients with HER2-positive advanced BTC and warrants further clinical trials in these patients. This study may provide a rationale for further investigations to clarify the efficacy and toxicity of trastuzumab-based combination chemotherapy in patients with HER2-positive advanced BTC.

## MATERIALS AND METHODS

### Human BTC cell lines

A total of 11 human BTC cell lines were used in this study. SNU-245, SNU-308, SNU-478, SNU-869, SNU-1079, SNU-1196, and SNU-2823 cell lines were obtained from Korean Cell Line Bank, Seoul, Korea. HuCCT1 and TFK-1 cell lines were obtained from RIKEN BioResource Center, Ibaraki, Japan. *HER2*-amplified human BTC cell lines, SNU-2670 and SNU-2773, were established from tumor tissues from BTC patients using a previously described method [31].

All the cell lines were maintained in RPMI-1640 culture media supplemented with 10% fetal bovine serum (WELGENE Inc., Gyeongsan, Korea) and 10 μg/mL gentamicin in a humidified atmosphere containing 5% CO_2_ at 37°C.

### Tested agents

Trastuzumab, a monoclonal antibody for HER2, was purchased from Genentech (South San Francisco, CA, USA). Dacomitinib (PF-00299804), an irreversible pan-HER inhibitor, and afatinib (BIBW-2992), a dual inhibitor of EGFR and HER2, were purchased from Selleck Chemicals LLC (Houston, TX, USA). Gemcitabine, cisplatin, and 5-fluorouracil (5-FU) were purchased from Lilly Korea Co., Seoul, Korea, JW Pharmaceutical Co., Seoul, Korea, and Ildong Pharmaceutical Co., Seoul, Korea, respectively.

### Cell growth inhibition assays

Cells were seeded at a density of 5,000 cells per well in 96-well plates and exposed to increasing concentrations of various targeted and cytotoxic agents for 72 h. After drug treatment, the absorbance of MTT dye was measured at 540 nm with a VersaMax microplate reader (Molecular Devices, Sunnyvale, CA, USA).

For colony formation assays, the cells were seeded at a density of 3,000 cells per well in six-well plates and treated with targeted agents. After 12 days, the cell colonies were stained with 0.1% Coomassie Blue solution (Sigma-Aldrich, St. Louis, MO, USA), and counted using a Gel Doc (Bio-Rad, Hercules, CA, USA). The data presented are representative of three independent experiments.

### Western blot analysis

Proteins were extracted and equal amount of proteins were separated on 10% sodium dodecyl sulfate polyacrylamide gels and transferred onto nitrocellulose membranes. The membranes were probed overnight at 4°C with appropriate primary antibodies. Primary antibodies against the following molecules were purchased from Cell Signaling Technology (Beverly, MA, USA): EGFR, phosphorylated EGFR (Tyr992), HER2, phosphorylated HER2 (Tyr1248), HER3, phosphorylated HER3 (Tyr1289), phosphorylated STAT3 (Tyr705), phosphorylated AKT (Ser473), phosphorylated ERK (Thr202/Tyr204), PARP, caspase-3, and caspase-7. Anti-α-tubulin and anti-β-actin antibodies were purchased from Sigma-Aldrich (St. Louis). Anti-cyclin A, anti-cyclin D, and anti-cyclin E antibodies were purchased from Santa Cruz Biotechnology (Dallas, TX, USA). Antibody binding was detected using an enhanced chemiluminescence system according to the manufacturer's protocol (Amersham Biosciences; Piscataway, NJ, USA). Anti-mouse and rabbit secondary antibodies were purchased from Thermo Scientific Inc. (Waltham, MA, USA).

### Cell cycle analysis

The cells treated with dacomitinib, afatinib, and trastuzumab at various concentrations for 48 h were harvested, fixed with cold 70% ethanol, and then stored at −20°C for at least 24 h. The cells were washed in phosphate-buffered saline (PBS) and incubated with 20 mg/mL RNase A (Invitrogen, Carlsbad, CA, USA) at 37°C for 20 min. Next, the cells were stained with 20 mg/mL propidium iodide (Sigma-Aldrich), and the DNA content of the cells (1.0 × 10^4^ cells per experimental group) was quantified using a FACS Calibur flow cytometer (BD Biosciences, Franklin Lakes, NJ, USA).

### *In vivo* study

Female Balb/c athymic nude mice aged 4–6 weeks were purchased from Central Lab Animal Inc., Seoul, Korea. The mice were injected subcutaneously in the right flank with 5 × 10^7^ SNU-2670 cells in 100 μL of PBS. After implantation of the tumor cells, the size of the resulting tumors was measured every other day using calipers; the body weight of each mouse was also determined every other day. The tumor volume was calculated using the following formula: (width^2^ × height)/2. When the tumor volume reached 150 to 200 mm^3^, the mice were randomly divided into four groups: control, trastuzumab, gemcitabine, and trastuzumab plus gemcitabine. Trastuzumab (4 mg/kg) and/or gemcitabine (100 mg/kg) was injected intraperitoneally twice a week for 3 weeks. At 37 days, the mice were euthanized with CO_2_. The tumors were excised and stored in liquid nitrogen until further analysis by IHC staining and western blot. The animal experiments were performed at the Biomedical Center for Animal Resource Development of Seoul National University, Seoul, Korea, according to the institutional guidelines.

### IHC of tumor tissues from xenograft model

The histologic sections from individual paraffin-embedded xenograft tumor tissues were deparaffinized and dehydrated. IHC detection of proliferating cells was conducted using anti-rabbit polyclonal antibody against Ki-67 (GeneTex, Inc., Irvine, CA, USA) at a dilution of 1:100. TUNEL assays were conducted for IHC detection of apoptosis using an ApopTag *In situ* Apoptosis Detection Kit (EMD Millipore, Billerica, MA, USA), in accordance with the manufacturer's protocol.

### Clinical materials

After informed consent was obtained, patients with unresectable or recurrent BTC who planned to undergo palliative chemotherapy were enrolled in the prospective cohort study to collect biomaterials for translational research. Data regarding patient demographics, pathologic classification, treatment response, PFS, and OS were obtained from medical record review. Treatment response was evaluated using a multi-detector CT scan by Response Evaluation Criteria in Solid Tumors version 1.1 [32]. PFS was calculated from the initiation of palliative chemotherapy to documented disease progression or death from any cause. OS was calculated from the start of chemotherapy to death from any cause.

HER2 positivity was defined as an intensity of 3+ by IHC or as a HER2/CEP17 (centromeric probe for chromosome 17) ratio of more than 2.0 by FISH, as previously described [4, 33–35].

### Statistics

Experimental data were expressed as the mean ± standard error (SE) and compared using Student's *t*-test. IC_50_ of chemotherapeutic agents was analyzed using SigmaPlot software (Systat Software, Inc., San Jose, CA, USA). The combined effects of various chemotherapeutic agents were analyzed by CalcuSyn software (Biosoft, Ferguson, MO, USA) using the Chou-Talalay method as previously described [36]. The CI values of < 1, 1 and > 1 indicate synergism, an additive effect, and antagonism, respectively. The median PFS and OS were calculated using the Kaplan–Meier method. Comparison of survival data was performed using the log rank test. All statistical tests were two-sided, with significance defined as *P* < 0.05. All analyses were performed using IBM SPSS version 22.0 (IBM, Armonk, NY, USA).

### Study approval

The animal experiments were approved by the institutional animal care and use committee (IACUC) of Seoul National University. Signed informed consent was obtained from each patient before study entry. This study protocol was reviewed and approved by the institutional review board (IRB) of the Seoul National University Hospital (IRB registration No. 1306-069-497) and conducted in accordance with the precepts established by the Helsinki Declaration.

## SUPPLEMENTARY MATERIALS FIGURES AND TABLES


