# Supplementary material for: Therapeutic implication of HER2 in advanced biliary tract cancer
Source: Oncotarget. 2016 Aug 9;7(36):58007–21. doi: 10.18632/oncotarget.11157 (PMC5295408; doi:10.18632/oncotarget.11157)
Supplement: Supplementary file 1 [file oncotarget-07-58007-s001.pdf]

Therapeutic implication of HER2 in advanced biliary tract cancer

Supplementary Materials

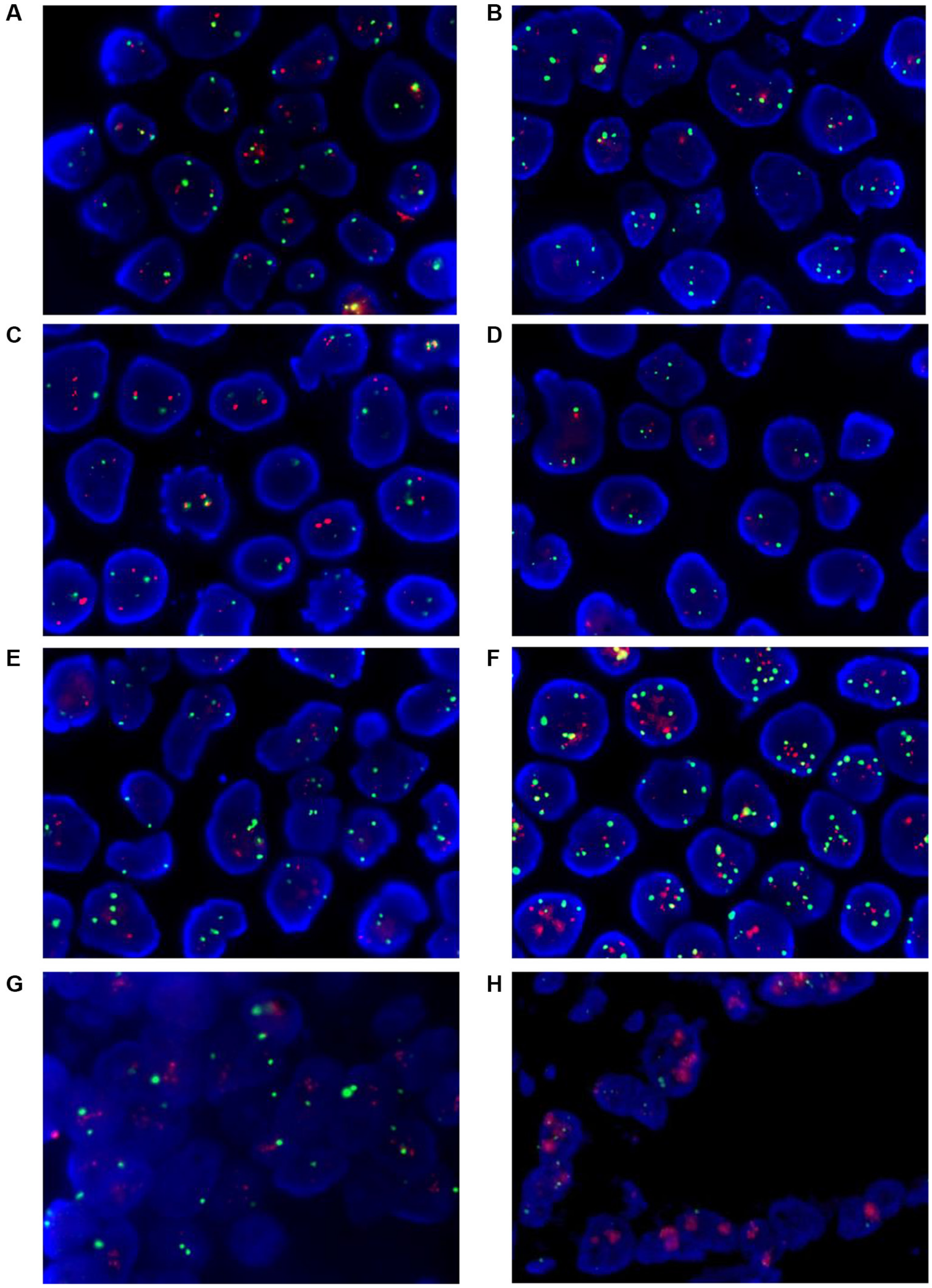

Supplementary Figure S1: *HER2* gene amplification status by fluorescent *in situ* hybridization (FISH). SNU-245 (**A**), SNU-308 (**B**), SNU-478 (**C**), SNU-869 (**D**), SNU-1179 (**E**), and SNU-1196 (**F**) cells did not harbor *HER2* amplification, while SNU-2670 (**G**) and SNU-2773 (**H**) were *HER2* FISH-positive. We determined HER2 positivity using the number of HER2 (red) and CEP17 (green) signals according to the ASCO/CAP guidelines (Wolff AC, *et al.* J Clin Oncol. 2013; 31:3997–4013).

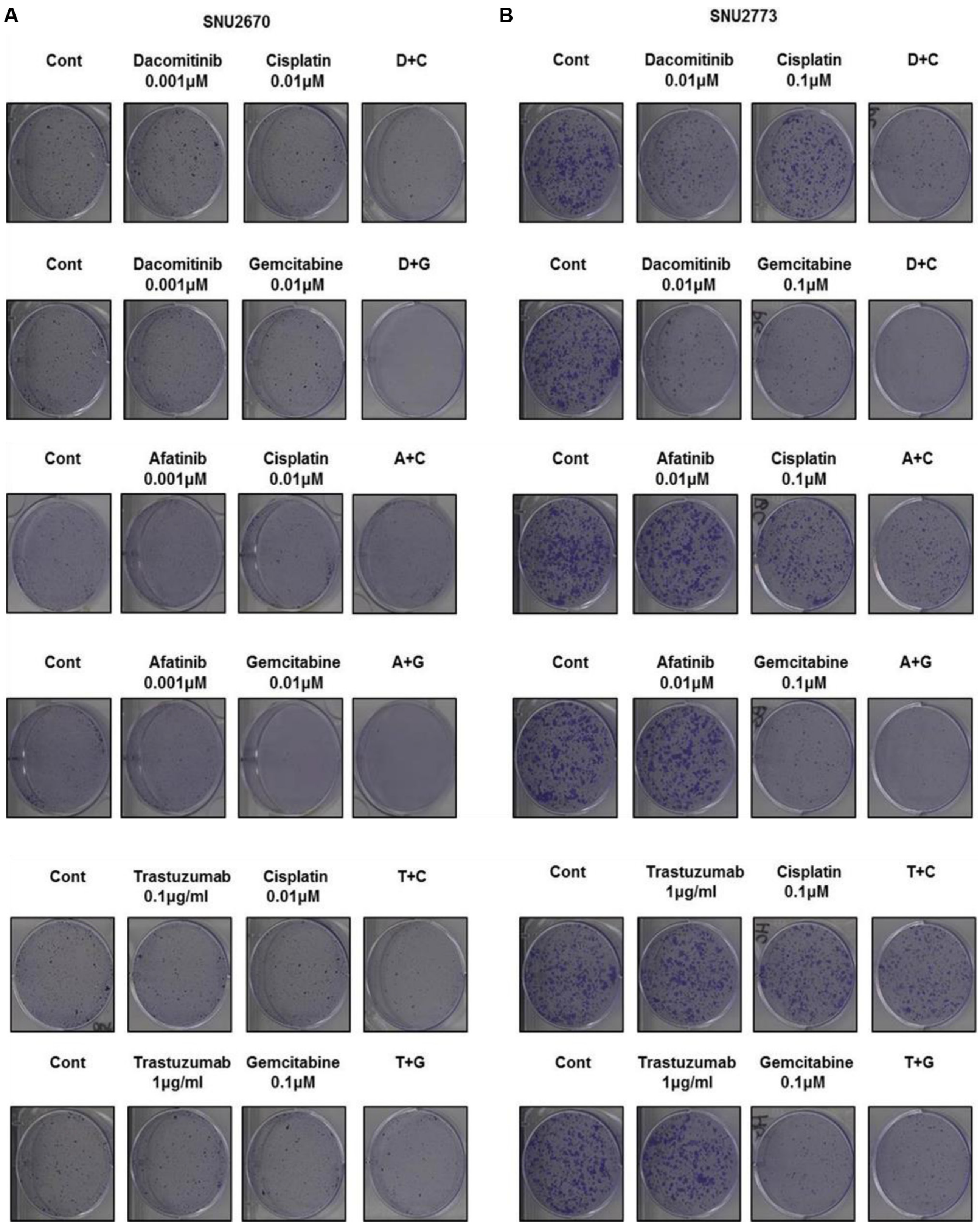

Supplementary Figure S2: Colony formation assays of combination treatment of a targeted agent and a cytotoxic agent. Combination of a targeted agent (dacomitinib, afatinib, or trastuzumab) and a cytotoxic agent (cisplatin or gemcitabine) demonstrated significantly decreased cell proliferation than a targeted agent alone in both SNU-2670 (**A**) and SNU-2773 (**B**) cells.

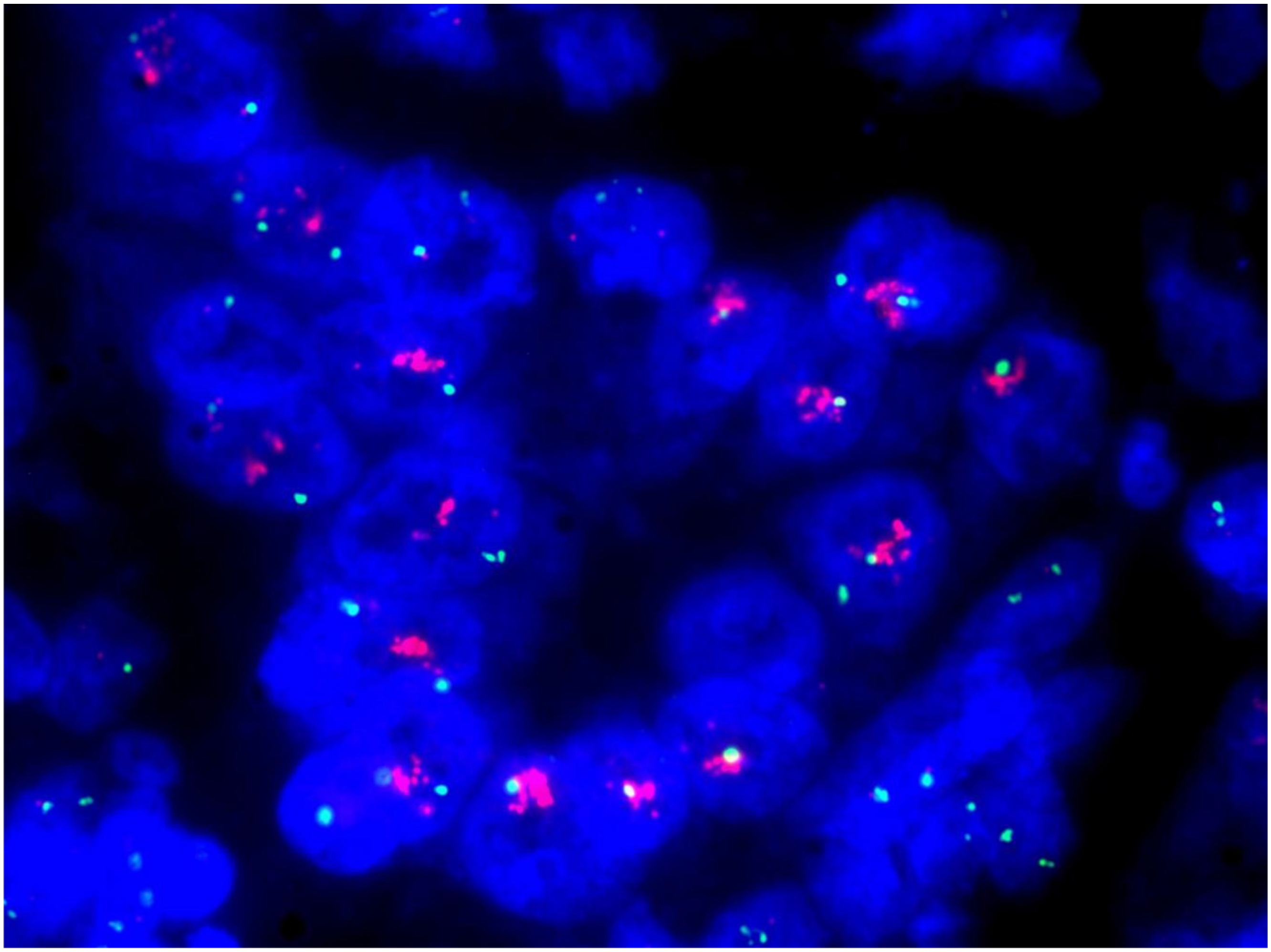

Supplementary Figure S3: *HER2* gene amplification status by FISH in patient C. In patient C, a biopsy specimen from metastatic periductal lymph nodes harbored *HER2* gene amplification with a HER2/CEP17 ratio of 9.53 by FISH. We determined HER2 positivity using the number of HER2 (red) and CEP17 (green) signals according to the ASCO/CAP guidelines (Wolff AC, *et al.* J Clin Oncol. 2013; 31:3997–4013).

Supplementary Table S1: Sensitivity of SNU-2670 cells to various targeted and cytotoxic agents

Drugs	Representative targets or mechanisms of action	IC_{50} (μM)
Dacomitinib (PF00299804)	EGFR, HER2, and HER4	0.10
Afatinib (BIBW2992)	EGFR and HER2	0.13
Gemcitabine	Pyrimidine analog	> 10
Cisplatin	Crosslinking of DNA	> 10
5-fluorouracil	Pyrimidine analog	> 10

Supplementary Table S2: Sensitivity of SNU-2773 cells to various targeted and cytotoxic agents

Drugs	Representative targets or mechanisms of action	IC_{50} (μM)
Dacomitinib (PF00299804)	EGFR, HER2, and HER4	1.50
Afatinib (BIBW2992)	EGFR and HER2	1.62
Gemcitabine	Pyrimidine analog	> 10
Cisplatin	Crosslinking of DNA	> 10
5-fluorouracil	Pyrimidine analog	>10
